# How do field of view and resolution affect the information content of panoramic scenes for visual navigation? A computational investigation

**DOI:** 10.1007/s00359-015-1052-1

**Published:** 2015-11-18

**Authors:** Antoine Wystrach, Alex Dewar, Andrew Philippides, Paul Graham

**Affiliations:** School of Informatics, University of Edinburgh, Edinburgh, UK; Centre for Computational Neuroscience and Robotics, University of Sussex, Brighton, UK

**Keywords:** View-based homing, Ants, Snapshot, Route navigation, Image matching

## Abstract

The visual systems of animals have to provide information to guide behaviour and the informational requirements of an animal’s behavioural repertoire are often reflected in its sensory system. For insects, this is often evident in the optical array of the compound eye. One behaviour that insects share with many animals is the use of learnt visual information for navigation. As ants are expert visual navigators it may be that their vision is optimised for navigation. Here we take a computational approach in asking how the details of the optical array influence the informational content of scenes used in simple view matching strategies for orientation. We find that robust orientation is best achieved with low-resolution visual information and a large field of view, similar to the optical properties seen for many ant species. A lower resolution allows for a trade-off between specificity and generalisation for stored views. Additionally, our simulations show that orientation performance increases if different portions of the visual field are considered as discrete visual sensors, each giving an independent directional estimate. This suggests that ants might benefit by processing information from their two eyes independently.

**Electronic supplementary material:**

The online version of this article (doi:10.1007/s00359-015-1052-1) contains supplementary material, which is available to authorized users.

## Introduction

There is enormous variation in the information provided by the visual systems of different animals (Land and Nilsson 2002). In general terms we can consider how eye design is driven by the developmental and metabolic cost of sensory apparatus and the informational requirements of an animal’s behavioural repertoire. Extracting greater volumes of sensory information (e.g. higher resolution or larger visual field) will always be costly (Snyder et al. 1977; Niven and Laughlin 2008) but these costs can be mitigated by specific adaptive value. A classic example from the insect kingdom is that male flies possess a small region of high acuity in their frontal visual field that facilitates precise mate chasing (e.g. Franceschini et al. 1981). The trade-off between metabolic cost and the value of the information provided is played out in the size of the high-resolution region. This raises the question as to whether the resolution of visual systems is always compromised between the metabolic cost and the inherent value of a higher visual resolution. An alternative is that low-resolution visual information is actually more useful for some visually guided behaviours.

We are interested in how vision relates to behaviour for view-based navigation, an orientation strategy shared by many species, from insects to humans (e.g. Wang and Spelke 2002; Wystrach and Graham 2012). This ability is particularly pronounced in the foragers of many social insects, in which individuals rapidly learn the visual cues required to guide their routes from nest to food, independently of other navigational strategies such as odour trails (Rosengren and Fortelius 1986; Harrison et al. 1989) or path integration (von Frisch 1967; Wehner et al. 1996). View-based navigation involves remembering egocentric views of the world from important locations (Cartwright and Collett 1983; Wehner and Räber 1979; Zeil 2012), a process for which we have good hypothetical models of how the visual information is used (Baddeley et al. 2012; Zeil 2012). Interestingly, view-based navigation specialists do not necessarily possess high visual resolution. For instance in ants, higher acuity can be seen in predatory species [e.g. *Gigantiops destructor* (Beugnon et al. 2001)], but not necessarily in the species that rely on vision predominantly for navigation [e.g. *Melophorus bagoti* (Schwarz et al. 2011)].

Here we take a computational approach in asking how visual resolution, field of view and the fact that ants have two eyes, influence the recovery of orientation using stored views in a simulation of complex environments that share many properties with the semi-arid habitats experienced by desert ants such as *Cataglyphis velox* (Mangan and Webb 2012) or *Melophorus bagoti* (Muser et al. 2005). We find that the coarse properties of desert ants’ eyes are well suited for parsimonious methods of visual route navigation.

## Methods

Simulations and analyses were performed using Matlab^®^ (MathWorks, Natick, MA, USA).

### Simulated world

**Fig. 1:**
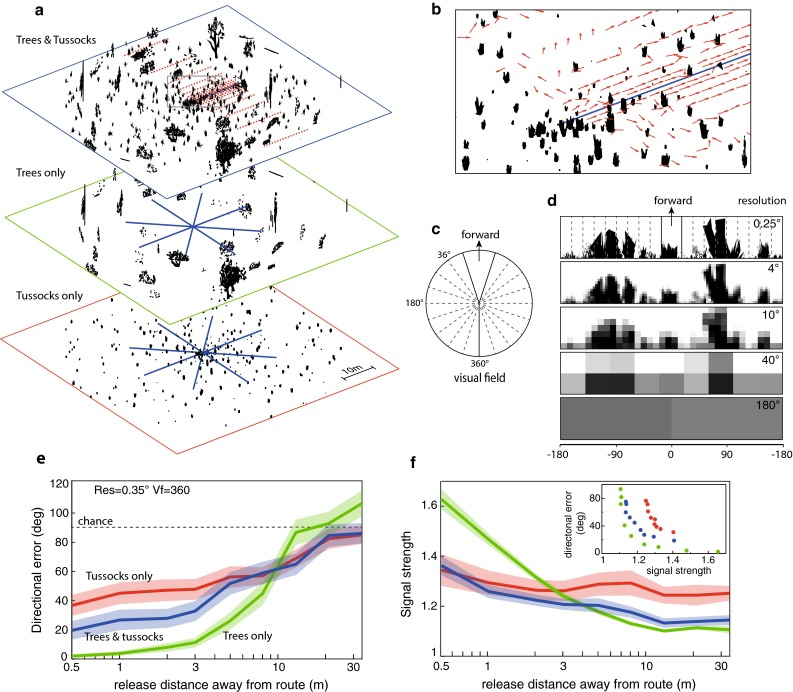
Simulating natural environments. **a** We generated six simulated worlds, two of each of three types: tussocks only (*bottom*); trees only (*middle*); trees and tussocks (*top*). Within each world we generated 8 training routes radiating from the centre of each world (*blue* *lines*). **b** Route performance was measured by asking how accurately could the route memories (given a particular eye design) be used to recover the route heading at different displacements from the route (*red dots* in **a** indicate release locations for one training route; red arrows in **b** indicate recovered headings at these locations). **c** The visual field was varied from 36° to 360° but always kept symmetrical about the forward facing direction. **d** Along with visual field, we co-varied resolution. Here, for the same scene, we show resolutions from 0.25°–180°. **e** The directional error (mean and 95 % confidence interval) is shown for locations at different distances from the training routes in each of the three world types: trees only (*green*); trees and tussocks (*blue*); and, tussocks only (*red*). Data presented here were collected from simulations with high resolution (0.35°) and a full visual field of 360°. The dashed line at 90° represents chance and the *x*-axis is non-linear to emphasise the region of interest. **f** For the same data as (**e**) we look at signal strength. Signal strength for a specific test location is defined as the degree of familiarity in the most familiar direction divided by the median familiarity from across all tested directions. The most familiar direction is that with the lowest value in the rIDF (see “Methods”). The graphs show mean signal strength (with 95 % CI) and the colours are as above. Inset shows directional error as a function of signal strength averaged for each release distance

The simulated worlds are generated in the same way as presented in Baddeley et al. (2012). The ‘worlds’ are inspired by the visually sparse, semi-arid habitats of *Melophorus bagoti* and consist of a random assortment of tussocks and trees (Fig. 1). Tussocks and trees are generated from sets of pre-defined black triangles in random configurations and are based on the scale of objects in *Melophorus’* environments, hence distances are given in metres. Tussocks are rendered as three-dimensional objects (~1 m in height), whereas trees are two dimensional as they are sufficiently far from the portion of the environments where testing was performed such that 3D was redundant.

### Training route and displacements

A 20-m-long training route is placed in the centre of the world from the nest to a fictive food site (blue line in Fig. 1a, top world). The simulated ant’s memory of this route is made up of 200 images taken at intervals of 0.1 m along this route with the views being limited by the particular resolution and field of view used in that iteration of the experiment. To test the algorithm, we used 17 discrete test positions 1 m apart along test transects that were parallel to the training route, at nine distances of 0.5, 1, 2, 3, 5, 8, 13, 21 or 34 m either side of the training route (red dots in Fig. 1a and origins of red arrows in Fig. 1b). For each of the two worlds and vegetation levels, the procedure was repeated for 8 routes radiating from the centre of the world (blue lines in Fig. 1a, two bottom worlds). Overall, this resulted in 306 test locations per training route (17 along each test route × 9 distances from the training route x 2 left/right displacements) and 2448 per world (306 × 8 training routes). Simulations were performed in three types of environment: tussocks only; trees only; trees and tussocks; with two different worlds generated for each type (Fig. S1). For each location we assess how well the simulated ant’s memory can be used to recover route appropriate direction. The results for different routes, environment types and worlds are combined to give the overall results.

Because the simulated worlds are bounded in size (to increase computational efficiency) there may be an interaction between very large displacements and the edges of the environment, such that for large displacements the majority of objects in the scene will fall on one half of the simulated ant’s retina. Although such bounded worlds still represent possible natural environments, they may not be typical and could skew the results. However, we observe that performance worsens with increased displacement at such a rate that any such confound will not influence our primary findings because interesting results are to be found for small to medium displacements.

### Derivation of heading

To test the effectiveness of stored route views for navigation, we ask whether those stored views can be used to recover appropriate orientations from the test locations. Test orientations are derived following the ‘Perfect Memory’ algorithm outlined in Baddeley et al. (2012) and more fully described in (Dewar et al. 2014). Briefly, for a particular test location we compare the current view with each image stored from the training route using the rotational image difference function, or rIDF (Zeil et al. 2003; Philippides et al. 2011). The rIDF is calculated by making multiple comparisons between a current and stored view using an image difference function, here the mean absolute difference between images:

$$d\left( {I,\;J,\;\theta } \right) = \frac{{\mathop \sum \nolimits \mathop \sum \nolimits \left| {I_{\text{m,n}} - J\left( \theta \right)_{\text{m,n}} } \right|}}{wh}$$

where *w* and *h* are image width and height, respectively, $I_{m,n}$ is the (*m*,*n*)th pixel of a stored view, *I*, oriented along the training route, and $J\left( \theta \right)_{m,n}$ is the (*m*,*n*)th pixel of current view, *J*, at a rotation of θ relative to the orientation of *I*. The estimate of the heading from the stored view, *I*, is then the orientation *θ* of the current view at which the rIDF, *d*, is minimised, that is:

$$m\left( {I,J} \right) = m\mathop {\text{in}}\limits_{\theta } d\left( {I,\;J,\;\theta } \right)$$

$$h\left( {I,\;J} \right) = {\text{arg m}}\mathop {\text{in}}\limits_{\theta } d\left( {I,\;J,\;\theta } \right)$$

To find the agent’s heading, minimum rIDF values, *m*(*I, J*), are calculated for each of the training views, and the heading, *h*(*I, J*), associated with the lowest minimum is selected.

We have selected what we consider a prime candidate model for visual route navigation but there are various ways in which animals might implement visual navigation. Within the insect navigation literature alone, there is a healthy debate regarding the algorithmic nature of visual guidance (Zeil 2012). We have chosen to simulate visual route navigation whereby an agent uses stored scenes to set a direction by aligning its body along the best matching orientation with its memories (Zeil et al. 2003; Graham et al. 2010) rather than by moving in a direction that reduces the mismatch between aligned current view with a single memory. Models of this type follow the so-called snapshot model (Cartwright and Collett 1983) and views act as attractors. While both styles of visual guidance have been implicated in ants (Collett 2010; Wystrach et al. 2012; Narendra et al. 2013), theoretical studies show that if natural scenes (as filtered through a particular visual system) contain information that is useful for one strategy, they will similarly contain information that can be used for the other (Zeil et al. 2003; Philippides et al. 2011). Therefore, our results have generality to snapshot-type models also.

### Signal strength

Using the model described, two types of information are directly available to an agent trying to recover its heading: The direction that matches best the training views and the quality of this match. We used a heuristic to approximate the signal/noise ratio and understand how match quality varies against directional error, namely, how much better the best matching direction is compared to the median match value across all directions (Fig. S2). Figure 1f shows that this signal strength measure tends to be inversely (but tightly) correlated with directional error and we thus focus on directional error in the results, although we note that measures of uncertainty are biologically important. For instance, they can be used to weigh the directions derived from view-based matching against other potentially conflicting directional cues such as from path integration (see for example Collett 2012; Legge et al. 2014).

### Visual system

From the simulation we create panoramic views that cover 360° in azimuth and 75° in elevation (starting from the horizon). These views are greyscale with black for objects, white for sky and grey where a pixel falls on the boundary of object and sky. Thus, different levels of grey reflect the proportions of sky/object covering a pixel.

We varied both resolution and azimuthal extent of the images. Azimuthal visual field varied from 36° to 360° in 10 steps of 36°. Resolution varied from 2 to 1024 azimuthal pixels (i.e. from 180° to 0.35°) in 10 steps increasing as 2^*n*^. Images were first obtained from the worlds at the highest resolution (0.35°) and visual field (360°), then subsampled at the desired resolution and finally trimmed to the desired visual field. The azimuthal centre of the image always corresponds to the forward facing direction (Fig. 1c, d) in the training views, i.e. along the training route. Because our investigation concerns bilaterian animals, the number of azimuthal pixels was even, constraining our views to a minimum of two pixels.

**Fig. 2:**
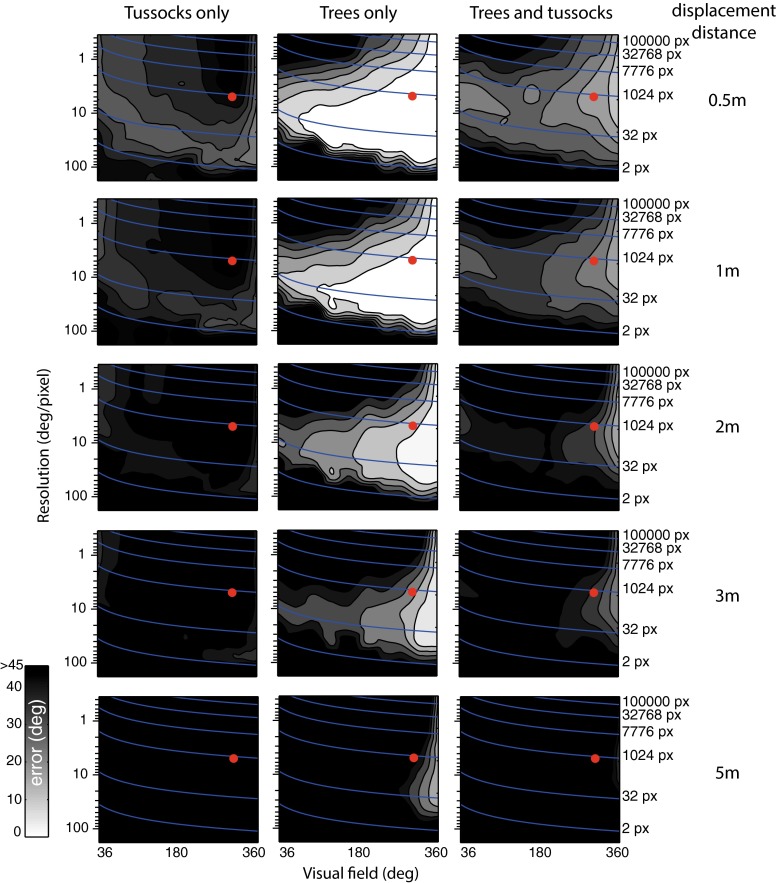
Performance as a function of resolution and azimuthal visual field. For the three world types (*columns*) we show how performance varies as a function of visual resolution and the azimuthal extent of the visual field. This analysis is repeated for release locations at different distances away from the training route (*rows*). In each panel grey levels represent mean directional error, with lighter shades meaning better performance. Errors have been interpolated from 10 visual field sizes ×10 resolutions regularly spaced on the maps (triangle-based cubic interpolation). Isolines are used to represent absolute number of pixels across resolution and visual field size.* Red dot* represents the visual field and resolution of *Melophorus bagoti* (Schwarz et al. 2011)

The approximate resolution for some well-studied ant navigators is around 5°, modelled here as 1 pixel covering 5° with a visual field of 300° (Schwarz et al. 2011; Zollikofer et al. 1995). This point in resolution visual field space is shown as a red dot in Fig. 2.

The combination of the simulated world and the eye model gives us views that capture the high-contrast boundary between objects and sky. This is likely to be particularly salient to any ant with UV green visual channels (Möller 2002) and mitigates against contrast problems caused by clouds and shadows. Indeed, in behavioural experiments this high-contrast boundary has been shown to be a sufficient substitute for a natural panorama (Graham and Cheng 2009a). Of course the sufficiency of this signal does not preclude other sources of visual information being important for ants, such as colour or texture. As yet we do not have detailed descriptions of the early stages of visual processing for ants and how this relates to visual navigation though we acknowledge that in future more detailed, ‘ground-truthed’ visual reconstruction models will be useful (see Narendra et al. 2013 and Stürzl et al. 2015 for progress).

### Sector matching

In the standard model, the current view is matched to stored views as a single whole image. However, it is possible to divide the visual field into subfields and to match each independently as if they were smaller views. To investigate the effect of such sector matching, we used a global visual field of 300° and a resolution of 5° as these parameters match the ant’s optics and fall within the optimal performance area given a single whole image (Fig. 2). We compare the performance obtained for a single sector, covering the frontal 300°, with two, three, four, five and six sectors. Thus, for a two-sector visual field, each sector is 150° wide and meet at the front of the ant, while for a three-sector visual field, each sector is 100° wide with the middle one centred on the frontal 100° and the others aligned contiguously on either side of the central sector, and so on for increasing numbers of even and odd sectors. To retrieve a single heading from these independent sectors, each sector is rotated to find the best matching heading with appropriately sized sectors of the stored route views centred on the training route direction. The final heading is the angular average of the headings across the sectors. Obviously for real ants the sectors would have to physically move together, and to implement this algorithm some form of working memory would be needed.

## Results

Our goal is to analyse visual navigation performance as a function of visual resolution and visual field properties. Within simulated worlds, we give a simulated agent training views from along a route. Performance is then determined by how successfully our algorithm can recover accurate route headings from locations that are near to, but away from, the training route. The average absolute error for a population of randomly selected headings would be 90°, varying between 180° (opposite to the correct direction) and 0° (towards the correct direction). Thus, we set 90° as the chance level (dashed line in Fig. 1e). As we showed above, this error measure is strongly associated with signal strength and thus gives a good intuitive performance metric. As a preliminary test we investigated performance for two replicates of each environment type, using a fixed set of visual parameters (visual field 360°; resolution 0.35°). The performance follows an intuitive pattern (Fig. 1e): firstly, the pattern of results was consistent across replicates, giving confidence that results are driven by general properties rather than specific environmental arrangements; secondly, performance deteriorates with an increasing distance of the test locations from the training route; and thirdly, distal objects are more useful than proximal objects in recovering a direction from an off-route location (Fig. 1e). This echoes the findings of Stürzl and Zeil (2007) who showed that when using a single stored snapshot for homing, the greater the average object distance, the greater the range of homing. So overall, these results provide a degree of validation for the properties of our simulated world and its interaction with a navigation algorithm. This allows us to confidently move onto our primary analysis.

### How visual field size and resolution influence navigation performance

Across all our simulated world types we ask how field of view and resolution influence performance. The pseudo-colour plots of Fig. 2 show the mean directional error for combinations of those parameters. This analysis is repeated for the different world types (columns) and for different distances away from the training route (rows). We can see that performance is influenced by both parameters (i.e. visual field and resolution). Generally, good performance (i.e. brighter areas in Fig. 2) is obtained for the largest visual fields, and smaller visual fields fail entirely for large displacements. This is an intuitive result, because smaller visual fields mean that large regions of the scene are ignored, thus increasing the likelihood of aliasing. More interestingly, we observe a compromise regarding resolution, with the best performance obtained for intermediate resolutions between 50° and 1°. Rather than resolution per se, this compromise may be about the number of pixels (with best results obtained for a range of one order of magnitude from 100 to 1000 pixels). To explain this by way of an example, when the agent has a smaller visual field it performs better with a higher resolution, presumably to mitigate against potential aliasing. Interestingly, the benefit of low resolution is pronounced for increased displacements from the training route. We return to this point in the discussion.

### How is navigational performance influenced using multiple subfields?

We here investigate the effect of dividing the visual field into several subfields that can be matched independently before an ultimate direction is chosen as the circular mean of the directions of each subfield’s best match. More biologically, this could be achieved for instance by keeping the information from both eyes, or both cerebral hemispheres, separate for visual matching, and then integrating both directions at a later stage. For this analysis, we used a global visual field of 300 degrees and resolution of 5° because these parameters match the resolution of well-studied ant foragers and fall within the optimal performance regions from Fig. 2. We separated this visual field into a number of subfields, up to a maximum of six.

**Fig. 3:**
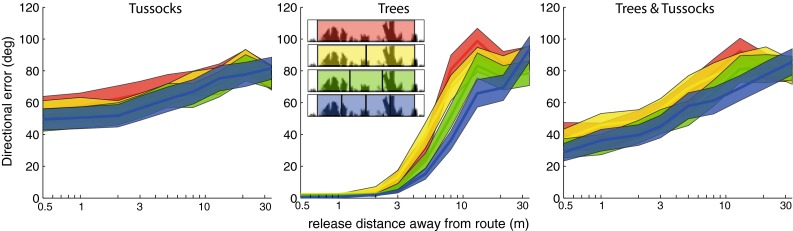
Performance as a function of number of visual subfields. For worlds containing tussocks, trees or trees and tussocks (left, middle and right, respectively) performance is shown when visual matching is undertaken using one (*red*), two (*yellow*), three (*green*) or four (*blue*) visual subfields across a range of release positions for a total visual field of 300° and resolution of 5°. Data shown are means with 95 % confidence intervals

Results show a general tendency for a higher number of subfields to improve performance (Fig. 3) with three other notable effects present in these data. First, the advantage of multiple subfields is subtle in the presence of tussocks, but clearly apparent in the ‘trees only’ environment (Fig. 3), suggesting that multiple subfields can help to overcome distortion in the perceived configuration of large distant objects (see “Discussion”). Second, the advantage of multiple subfields is maximal for intermediate release distances away from the training route as small or large displacements lead to a ceiling (performance always high) or floor effect (performance always low), respectively (Fig. S3). Finally, there is no trend for a continued increase in performance for added subfields (Fig. 3 and additional data not shown). Performance seems here to improve up to four subfields, but not beyond.

## Discussion

We have presented the results of a series of simulations investigating how the visual field and resolution of a visual system influence the efficacy of simple visual orientation strategy. Our primary finding is that for an agent with wide-field vision, a lower visual resolution is better suited to recovery of a route direction from the minimum in an rIDF. This is manifest in the improved performance for low-resolution systems when trying to recover route headings from off-route locations. So we can see that, as one moves away from the familiar route, if there is still information for homing this is likely to reside in lower spatial frequencies. At these off-route locations a higher resolution sensor might lose performance because objects of small apparent size are more likely to give an ambiguous high spatial frequency signal. Therefore, for a given environment, the ideal resolution will represent a trade-off between accurate recognition and range of use, thus balancing the specificity of a stored scene (how precisely a scene describes a specific location) with the distance over which the scene’s navigational information is useful. Given the limitations of our simulation and the different requirements for on- and off-route navigation it is hard to provide a specific figure for the ideal resolution for visual navigation, but it may well be lower than observed in ants that are visual navigation specialists (e.g. *Melophorus bagoti*, Schwarz et al. 2011).

That there is information in low-resolution scenes is demonstrated by studies in autonomous navigation. In an automotive task, Milford (2013) asked a car navigation system to localise itself within a previously learnt route. When using very low-resolution versions of panoramic scenes (even 4 × 4 pixels) to represent the familiar route, the algorithm could localise accurately when temporal information was used to mitigate against ambiguities. In a more biologically relevant robot task, Stürzl and Mallot (2006) showed that initially using only low-frequency components of a visual image and then iteratively matching higher frequencies as the robot gets closer to a discrete goal, allows it to home from a larger region than if visual matching is performed with views containing all spatial frequencies. Along with our results, we can see how for some navigational tasks low-resolution visual systems can perform better than high-resolution visual systems; that is, navigational performance can be increased despite reducing the amount of information and low-resolution vision need not always be framed as a trade-off between cost and performance. For some tasks, low-resolution can lead directly to higher performance.

### Are two eyes better than one?

We additionally find that performance can be improved if the agent is given multiple discrete visual wide-field sensors and matches those to stored views independently. This is an interesting result as it suggests that there may be an improvement to visual navigation if animals independently match the scenes experienced by each eye. To illustrate this idea, imagine a world with two trees some metres from the agent: a tall poplar projecting onto the left eye and a wide hazel tree projecting onto the right during training. When the agent is displaced, the perceived shape of such objects is largely conserved (i.e. the hazel and poplar tree are still perceived as a wide blob and tall shape, respectively), but the perceived inter-object angles (i.e. the angle between the two trees on the retina) can be quite different when viewed from the new vantage point. If the agent attempts to match the whole visual field at once, it will be unable to match both shapes simultaneously as when one tree matches the training memory, the other will not fit, resulting in two directions that provide a mediocre overall match. However, if the agent processes the information from its two eyes independently, each eye will recover a decent match (essentially matching the correct tree) for different directions which can be subsequently combined to set an average direction based on both trees. By breaking down the visual field into smaller subfields, each would be more concerned by individual shapes and less by inter-object configuration, the agent may then be able to recover good directional information despite large perceived distortion. The increased importance of shape would be an indirect consequence of the size of the subfield and not as a result of any object recognition mechanisms (see above).

### How generalizable are these results?

Of course in reality, the visual requirements of a task such as navigation extend beyond the coarse properties of the eye. The temporal and spectral tuning of photoreceptors will have to match the natural image statistics experienced by real ants in their natural habitat. We also did not consider how a non-uniform visual array (Land and Nilsson 2002) might influence navigational performance. At the moment, our simulation only represents an idealised visual system that extracts contrast boundaries without error. Further research into the visual system of ants (e.g. Ogawa et al. 2015), allied to more realistic simulations (e.g. Stürzl et al. 2015), will enable consideration of more nuanced issues.

A second concern is that navigation may be based on further visual processes where the initial (raw) visual input is used to identify specific visual objects in the world (Cartwright and Collett 1983) rather than being used as a raw holistic array (Zeil et al. 2003). The prevalent view is that object recognition and labelling is unnecessary given the inherent information available in a panoramic array (Zeil et al. 2003; Philippides et al. 2011; Figs. 1, 2, 3 here) and there is a growing set of circumstantial evidence from behavioural studies where the performance of ants seems not to be based in the identification of specific natural (Graham and Cheng 2009b; Wystrach et al. 2011b; Zeil et al. 2014) or artificial (Wystrach et al. 2011a) objects. However, this is not to say that top-down processes are not at play during visual navigation in insects. Recent studies of flies (van Swinderen 2007) and bees (Paulk et al. 2014) introduce the idea of visual attention in insects which might allow for a flexible weighting of different areas of the visual field.

## Conclusion

We have shown that the properties of some ant eyes (wide field and low resolution) may be ideal for some types of visual navigation and can perform better than would higher resolution visual systems. In summary, this suggests that low-resolution vision is not always a compromise of performance against cost. For our simulated agents low-resolution vision was beneficial for navigation, the question is raised as to whether this is true for other animals. One intriguing example is that of box jellyfish who display visual navigation based on terrestrial cues as perceived through Snell’s window. These animals possess lenses that focus light accurately but they have shifted their retina away from the focal point, thus blurring the image perceived (Garm et al. 2011; Nilsson et al. 2005). For humans, scene recognition is dependent on information from wide-field panoramic scenes (Epstein 2008) and as the visual periphery in humans is low resolution, it may be that low-resolution information is also used for some spatial tasks, whereas other tasks obviously rely on information from the high-resolution fovea.

## Electronic supplementary material

Supplementary material 1 (PDF 358 kb)

Supplementary material 2 (PDF 98 kb)

Supplementary material 3 (PDF 132 kb)

Supplementary material 4 (DOCX 14 kb)
