# Supplementary figures and images for: How do field of view and resolution affect the information content of panoramic scenes for visual navigation? A computational investigation
Source: J Comp Physiol A Neuroethol Sens Neural Behav Physiol. 2015 Nov 18;202:87–95. doi: 10.1007/s00359-015-1052-1 (PMC4722065; doi:10.1007/s00359-015-1052-1)

# WORLD 1

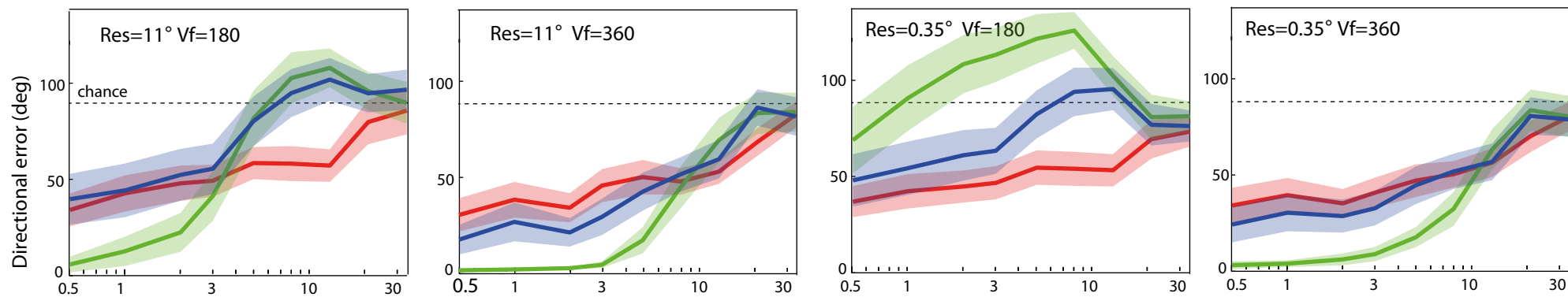

# WORLD 2

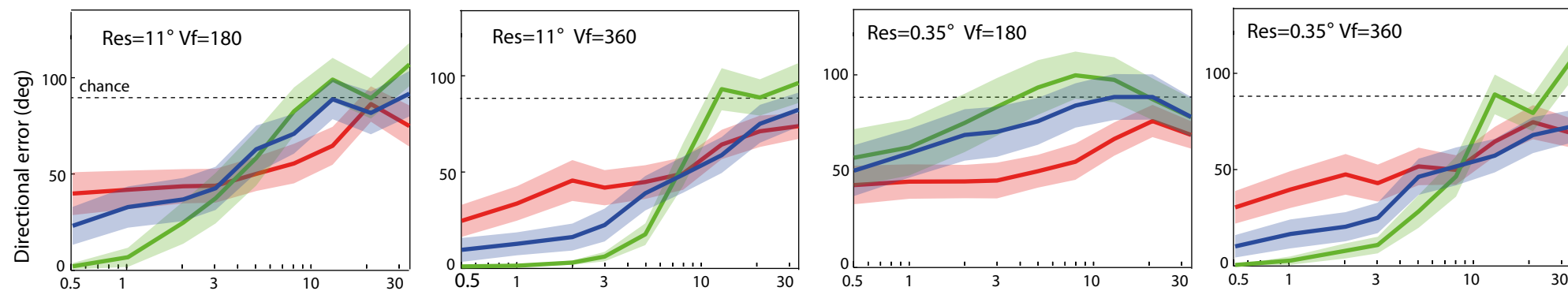

# BOTH WORLDS

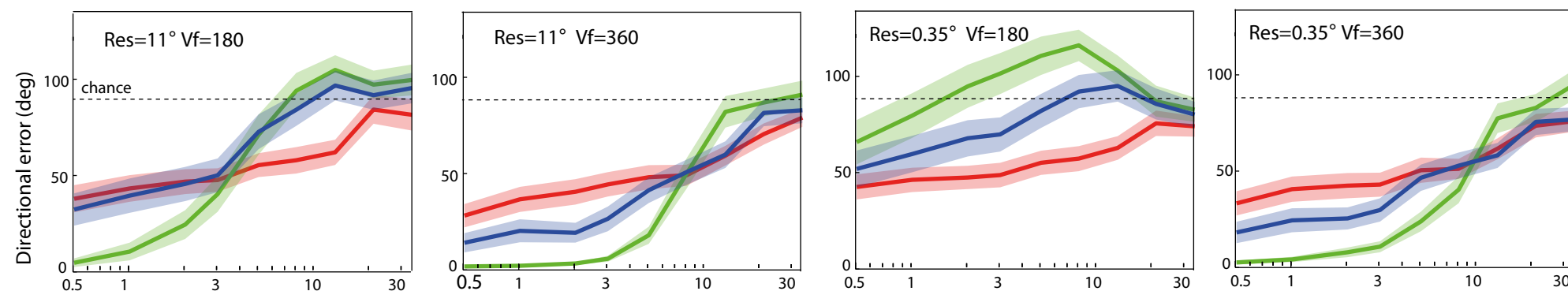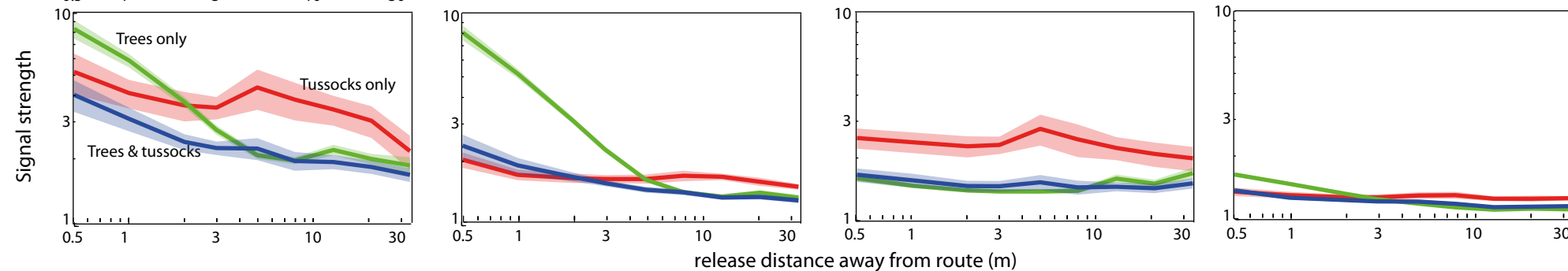

Supplement: Supplementary file 1 — Supplementary material 1 (PDF 358 kb) [file 359_2015_1052_MOESM1_ESM.pdf]

Percentage of mismatch

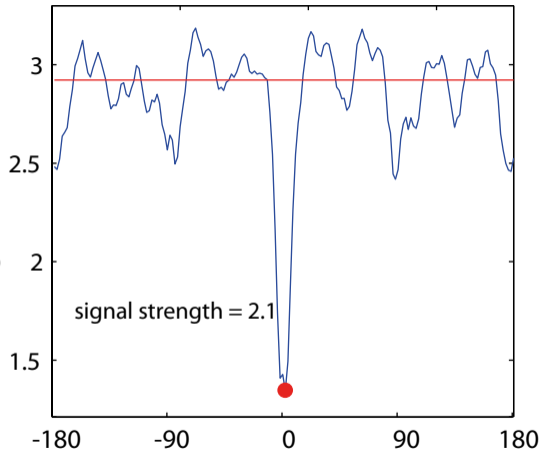

Degrees away from correct direction

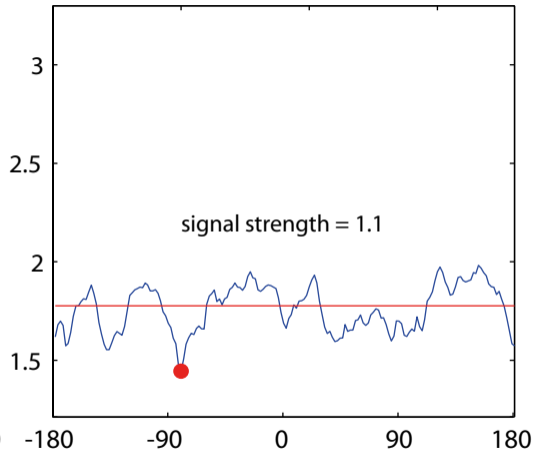

Supplement: Supplementary file 2 — Supplementary material 2 (PDF 98 kb) [file 359_2015_1052_MOESM2_ESM.pdf]

visual field = 300°    visual field = 120°    visual field = 60°

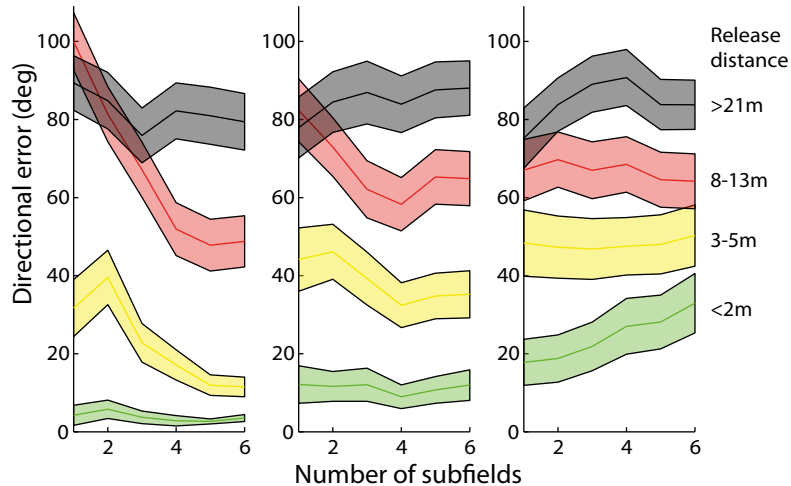

Supplement: Supplementary file 3 — Supplementary material 3 (PDF 132 kb) [file 359_2015_1052_MOESM3_ESM.pdf]
