# Supplementary material for: How do field of view and resolution affect the information content of panoramic scenes for visual navigation? A computational investigation
Source: J Comp Physiol A Neuroethol Sens Neural Behav Physiol. 2015 Nov 18;202:87–95. doi: 10.1007/s00359-015-1052-1 (PMC4722065; doi:10.1007/s00359-015-1052-1)
Supplement: Supplementary file 4 — Supplementary material 4 (DOCX 14 kb) [file 359_2015_1052_MOESM4_ESM.docx]

**Fig. S1. Consistency across worlds**. The directional error (mean and 95% confidence interval) is shown for locations at different distances from the training routes in each of the three world types: Trees only (green); Trees and tussocks (blue); and, tussocks only (red). Data presented here was collected from simulations for two different resolutions (11° and 0.35°) and for two visual field size (180° and 360°). The dash line at 90° represents chance and the x-axis is non-linear to emphasise the region of interest. Results are shown for world 1 only (**Row 1**), World 2 only (**Row** **2**), and both Worlds (**Row 3**). **Row 4:** Signal strength for both Worlds. (see Fig. S2 for explanation of signal strength). The graphs show mean signal strength (with 95% CI) and the colours are as above. Note that higher resolution leads to lower signal strength. This is because a higher ratio of pixels stay fully white (sky only) or black (tussocks only) and thus may not contribute to differences across facing directions.

**Fig. S2. Image differences and signal strength**. Image difference functions between a training location and two test locations: a small displacement (left panel) and a large displacement (right panel). The red dot highlights the most familiar direction (i.e. minimum mismatch) and red line indicates the median mismatch. Signal strength is calculated as median/minimum mismatch. Note that even though the match values are roughly similar for both test locations, signal strength is better for the smaller displacement (left panel).

**Fig. S3. Multiple subfields and visual field size.** Effect of dividing the visual field into multiple subfields across a range of displacement distances (Green: <2m; Yellow:3-5m; Red:8-13m and Black:>20m) and visual field sizes (left: 300°; middle: 120° ; right: 60°). Data shown is mean with 95% confidence intervals. Note that multiple subfields are most beneficial for medium displacements (3-5m and 8-13m) and become deleterious with a small visual field (60°).
